# Genital warts and inflammatory bowel diseases: Danish real‐world evidence to assess patient‐relevant outcomes

**DOI:** 10.1002/ueg2.12223

**Published:** 2022-03-23

**Authors:** Paul McLellan, Julien Kirchgesner

**Affiliations:** ^1^ Department of Gastroenterology AP‐HP, Hôpital Saint‐Antoine Paris France; ^2^ Department of Gastroenterology Sorbonne Université, INSERM, Institut Pierre Louis d’Epidémiologie et de Santé Publique, AP‐HP, Hôpital Saint‐Antoine Paris France

Inflammatory bowel diseases (IBD) are associated with an increased risk of various complications related to human papilloma virus (HPV), including cervical neoplasia and anal squamous cell carcinomas.^1^, ^2^ This risk may be mainly driven by thiopurines, as it promotes primarily viral infections. While thiopurines have been associated with an increased risk of skin warts in patients with IBD followed in tertiary care centers,^3^ the risk of genital warts had never been assessed in a population‐based cohort of patients with IBD. Although a benign disease, the burden of genital warts is substantial with a physical and psychological impact. Gastroenterologists are marginally involved in the therapeutic management, as local treatments are prescribed in first line by the general practitioner or dermatologist. HPV immunization through vaccination is available since 2006 and is effective against HPV serotypes 6 and 11, which are mostly involved in the development of genital warts. Finally, the real burden remains difficult to assess due to underreporting by patients and doctors.^4^

In the current issue of the *United European Gastroenterology Journal*, Elmahdi et al. assessed the risk of developing genital warts in patients with IBD using a nationwide population‐based cohort from 1996 to 2018.^5^

Using Danish registries, 49,163 IBD patients were matched by age, sex, and HPV immunization status to 491,665 individuals. They observed a 33% increased risk of genital warts in patients with IBD compared to the matched population (HR, 1.33 [95% CI: 1.19–1.49]). Compared to the general population, the excess of risk related to IBD was mainly observed in women (HR, 1.54 [95% CI 1.33–0.79]). Patients with Crohn's disease were at increased risk compared to patients with ulcerative colitis after adjusting for treatment (HR, 1.13 [95% CI 1.01–1.27]), and patients exposed to thiopurines were particularly at excess risk (HR, 1.50 [95% CI 1.34–1.67]). Some limitations need to be acknowledged. Treatment exposure was based on the maximal treatment until further escalation during follow‐up, which could lead to treatment misclassification. Smoking status was not collected and residual confounding could not be excluded. HPV vaccinated patients were excluded and it would have been interesting to assess the effectiveness of the vaccination campaign in preventing genital warts in patients with IBD. Nevertheless, this study is of great value compared to the available literature.

Danish health registries have been used for many years to address key clinical questions in the field of IBD.^6^, ^7^, ^8^ One of the main strengths of this data source is the big sample size, which allows to assess the risk of outcomes with low incidence and the impact of therapeutic interventions in a subset of patients. Recently, the effectiveness and safety of concomitant administration of allopurinol and thiopurines has been assessed in more than 10,000 patients with IBD treated with thiopurines. Overall, 2.7% of patients concomitantly received allopurinol and no increased risk of adverse events was identified.^9^ Despite many strengths, this source of real‐world data has some limitations, notably related to the non‐collection of environmental factors, clinical symptoms, and biological results. In comparison, prospective dedicated inception cohorts assess these parameters notably effectiveness outcomes, but it comes with higher cost and time‐to‐completion compared to administrative healthcare databases. The use of aminosalicylates remains controversial in Crohn's disease and Burisch et al assessed its impact on Crohn's disease course based on an inception cohort of patients with IBD, the Epi‐IBD cohort.^10^ They reported a quiescent disease course without need of additional treatment during follow‐up in a substantial group of patients with Crohn's disease treated with aminosalicylates. These studies highlight the potential of real‐word evidence derived from real‐word data to assess patient‐relevant outcomes.

In conclusion, Elmahdi et al. provides new insights on the risk of genital warts in patients with IBD, who are particularly at risk of genital neoplastic lesions. Based on the Danish registries and using a sound methodology, they concluded to a 33% increased risk of genital warts in patients with IBD compared to the general population. Systematic cervical screening should be performed according to national guidelines and gastroenterologists should be aware of the risk of genital warts, notably in women with IBD treated with thiopurines. Finally, HPV vaccination is a key element to prevent genital warts and must be recommended in patients with IBD according to national guidelines.

## Data Availability

There is no specific data included.
